# Direct scavenging of ROS by S-PPE NP reduces oxidative stress-induced stimulation of the SESN2/AMPK/KIM-1 pathway in acute kidney injury

**DOI:** 10.1080/0886022X.2025.2509802

**Published:** 2025-06-08

**Authors:** Chen Jiao, Hengyi Li, Yongdong Wu, Lemei Hu, Fengzhang Huang, Ming Liang

**Affiliations:** a Department of Nephrology, the Second Affiliated Hospital, School of Medicine, South China University of Technology, Guangzhou, PR China; b School of Medicine, South China University of Technology, Guangzhou, PR China; c Department of Nephrology, Guangzhou First People’s Hospital, Guangzhou, PR China

**Keywords:** Acute kidney injury, oxidative stress, ischemia–reperfusion injury, sens2

## Abstract

**Background:**

Previous research demonstrated that treatment of acute kidney injury (AKI) with the antioxidant S-PPE NP reduced the levels of the oxidative stress-responsive protein Sestrin2 (SESN2), and suggested that kidney injury molecule 1 (KIM-1) could serve as a biomarker for early tubular injury. A comprehensive elucidation of the regulatory effects of S-PPE NP on SESN2 and KIM-1 expression in ischemia-reperfusion injury-AKI (IRI-AKI) could enhance therapeutic approaches for AKI.

**Materials and methods:**

An *in vitro* human kidney-2 (HK-2) cell hypoxia/reoxygenation (H/R) model and a mouse IRI model were utilized at various time points to assess the expression of SESN2 and KIM-1 and to evaluate the impact of S-PPE NP treatment. The functionality of the SESN2/AMPK/KIM-1 signaling pathway was also confirmed.

**Results:**

Significant upregulation of SESN2 and KIM-1 was observed in both H/R and IRI models, which was attenuated following S-PPE NP treatment. Overexpression of SESN2 resulted in enhanced AMPK phosphorylation and reduced KIM-1 levels, whereas inhibition of AMPK phosphorylation with compound C did not affect SESN2 levels but led to an increase in KIM-1 levels.

**Conclusion:**

SESN2 serves as a protective factor in the initial phase of renal IRI-AKI, facilitating renal repair by promoting AMPK phosphorylation, which subsequently suppresses KIM-1 expression. Moreover, S-PPE NPs effectively mitigate IRI-AKI by directly scavenging reactive oxygen species and reducing SESN2 expression.

## 1. Introduction

Renal ischemia-reperfusion injury (IRI) occurs when blood flow to the kidney is temporarily interrupted and subsequently restored. This injury activates pathways involving oxidative stress, inflammation, and cell death, potentially leading to acute renal dysfunction and failure. The severity of renal IRI is influenced by the duration and extent of the ischemic injury [1]. Current therapeutic approaches primarily involve supportive care and management of complications, with few treatment options available [2].

It is crucial to explore novel interventions for treating AKI. At present, various inorganic nanomaterials with reactive oxygen species (ROS)-scavenging capabilities have been investigated for reducing oxidative damage associated with AKI, by emulating antioxidant enzyme-like activities and reacting with ROS. In contrast, the application of polymeric nanomaterials in AKI management remains an underexplored research area. Recently, ROS-sensitive chemical bonds, such as thioethers and thiols, have been extensively incorporated into polymer structures, enabling direct ROS scavenging for effective AKI treatment, rather than targeting oxidative stress directly [3–7], rather than directly interfering with oxidative stress targets (e.g., NOX4, GPX3, MSRA, GJB2, STC2, SESN2, NRF2, ACOX2, MAPT, HMOX1, AIFM1, etc.).

In this context, our laboratory designed and synthesized hyperbranched polyphosphonates (PPEs) containing sulfide linkages (S-PPE NPs). These nanoparticles demonstrate efficient uptake and scavenging capacities for various ROS, which are oxidized by sulfide into sulfone or sulfoxide. Preliminary findings indicated that treatment with S-PPE NP reduced the expression of the oxidative stress-sensitive protein SESN2 following glycerol-induced AKI. Additionally, a decrease in the expression of the kidney injury molecule 1 (KIM-1) was observed, implicating SESN2 in the response to AKI treatment with S-PPE NP [8]. However, the specific mechanisms remain unclear and warrant further investigation. SESN2, a stress-responsive protein, mitigates oxidative stress and enhances mitochondrial response to IRI [9–12]. For instance, in cardiac IRI, SESN2 facilitates AMPK activation through the LKB1-AMPK pathway during ischemic injury [13]. A thorough exploration of how S-PPE NP influences SESN2 expression and the biomarker KIM-1 in IRI-AKI could enhance therapeutic strategies for AKI.

## 2. Materials and methods

All animal procedures followed pertinent legislation and institutional guidelines and obtained the approval of the Medical Ethics Committee of South China University of Technology.

### 2.1. Data collection and clustering of RNA sequencing datasets

The Gene Expression Omnibus (GEO; https://www.ncbi.nlm.nih.gov/gds/) is a public database used for storing, accessing and processing a large scale of gene expression data for microarray datasets and high-throughput genomics datasets. For identifying differentially expressed genes (DEGs) in ischemic AKI, we analyzed the RNA sequencing dataset GSE142077 [14] which includes samples from human preischemic, ischemic, and ischemia–reperfusion kidneys. In this study, the R statistical language (version 4.2.1) was applied to the probe matching and the normalization process. Using preischemic as a normal control, we compared the DEGs in ischemic mesangial, ischemic-reperfused, and preischemic kidneys in the RNA sequencing dataset *via* the EdgeR package [15]. The filtering criteria for DEGs were set at |log2(FoldChange)| > 1 and an adjusted *p* value < 0.05. The web-based software Hiplot (https://hiplot.com.cn/) was employed for the generation of volcano plots.

### 2.2. Functional and pathway enrichment analyses

The Database for Annotation, Visualization, and Integrated Discovery (DAVID, david.ncifcrf.gov/) provides systematic and all-sided functional annotation resources available, which permit the investigation of the biological significance of extensive gene datasets. DEGs underwent GO and KEGG pathway enrichment analyses (*p* < 0.05), with the analysis results being visualized using the web-based software HiPlot. Furthermore, Genes underwent a GSEA under the assistance of the R package ‘clusterProfiler’. Genes with a *p* < 0.05 and a false discovery rate (FDR) < 0.25 were confirmed to present significant difference. The results of the enrichment analysis pertaining to SESN2 were subsequently filtered and visualized using the R package ‘ggplot2’.

### 2.3. Cell culture, hypoxia/reoxygenation (H/R) model, apoptosis, and SESN2 protein expression

The HK-2 cell lines (American Type Culture Collection, ATCC, Manassas, USA) were cultured in our laboratory in Dulbecco’s modified Eagle’s medium/nutrient mixture F-12 (DMEM/F12; Gibco, Thermo Fisher Scientific, USA) supplemented with 10% fetal bovine serum (FBS; Gibco) at 37 °C in a 5% CO_2_ atmosphere, in accordance with standard laboratory practice. The *in vitro* H/R model was established. After being subjected to a nutrient-free (devoid of glucose and serum) hypoxic environment (5% CO_2_, 1% O_2_, and 94% N_2_) for a period of 6 h, cells underwent 8 h of reoxygenation (5% CO_2_, 21% O_2_, and 74% N2) [16]. The HK-2 cells fell into four groups: (1) a control group maintained in a nutrient-sufficient, normoxic environment; (2) the S-PPE NP group that did not receive any additional treatment; (3) the H/R group without any additional treatment; and (4) the S-PPE NP + H/R group. Apoptosis was assessed *via* an Annexin V-FITC kit (Beyotime, C1062S), and the protein and mRNA expression levels of SESN2 and kidney injury molecule 1 (KIM-1) were subjected to Western blotting and real-time quantitative polymerase chain reaction (RT-qPCR) analyses, respectively.

### 2.4. Transfection

Suzhou GenePharma (Jiangsu, China) took charge of designing the small interfering RNA (siRNA) that targeted the human SESN2 mRNA, along with its relevant negative control (NC), and the negative control (NC). The sequences for si-SESN2 were:

SESN2-Homo650: 5′-CACCCUGACUACUUUUACCATT-3′ and

5′-UGGUAAAGUAGUCAGGGUGTT-3′;

SESN2-Homo1655: 5′ -CUCAAGGUCUAUUAUCAAGATT-3′ and

5′-UCUUGAUAUAGACCUUGAGTT-3′;

SESN2-Homo1703: 5′-CGAAGAAUGUACAACCUCUTT-3′ and

5′- AGAGGUUGUACAUUCUUCGTT-3′;

SESN2-Homo2141: 5′-GCUCGGAAUUAAUUGUGCCATT-3′ and

5′-UGGCACAUUAAUUUCCGAGCTT-3′.

The sequences for si-NC were: 5′- UUCUUCCGAACGUGUCACGUTT -3′ and 5′-ACGUGACACGUGUCGGGAGAATT -3′. The SESN2 overexpression plasmid targeting human SESN2 and the corresponding NC was designed by Suzhou GenePharma Co., Ltd. (Jiangsu, China).

10 μmol/L siRNAs and SESN2 overexpression were introduced into HK-2 cells *via* Lipofectamine^®^ RNAiMAX (Invitrogen, Thermo Fisher Scientific, USA) as per the producer’s protocol. In brief, once the cells had reached 60–70% confluence, the siRNAs combined with Lipofectamine^®^ RNAiMAX were diluted in Opti-MEM (free of antibiotics and FBS) to receive 6 h of incubation. RT–qPCR and western blotting served for assessing the transfection efficiency 24 h and 48 h posttransfection, respectively. The si-SESN2 with the highest silencing efficiency was identified. Following a 48-h transfection period, HK-2 cells were treated under hypoxia and reoxygenation condition. The HK-2 cells fell into six groups: (1) a control group under nutrient-sufficient, normoxic conditions; (2) the H/R group without treatmentthe S-PPE NP group, the control group receiving S-PPE NP treatment; (3) the si-NC+H/R group, the H/R group transfected with si-NC; (4) the si-SESN2 + H/R group, the H/R group transfected with si-SESN2; (5)the OE-NC+H/R group, the H/R group transfected with OE-NC; (6)the OEi-SESN2 + H/R group, the H/R group transfected with OE-SESN2. Prior to S-PPE NP+H/R treatment, HK-2 cells were transfected with the human SESN2 overexpression plasmid and siRNAs. These transfected cells fell into eight groups: (1) a control group under nutrient-sufficient, normoxic conditions; (2) an S-PPE NP group without any treatment; (3) an H/R group without any treatment; (4) an S-PPE NP+H/R group; (5) an si-NC+S-PPE NP+H/R group; (6) an OE-NC+S-PPE NP+H/R group; (7) an OE-SESN2 + S-PPE NP+H/R group. After the quantification of intracellular ROS level with the 2′−7′-dichlorodihydrofluorescein diacetate (DCFH-DA) probe (Beyotime), the solution was diluted 1:1000 as per the producer’s protocol. The cells added with a proper amount of the dilluted DCFH-DA solution received half an hour of incubation in the dark. Subsequently, the ROS levels were observed *via* an inverted fluorescence microscope.

### 2.5. Construction of an animal model of renal IRI

Healthy, specific pathogen-free (SPF) male C57BL/6J mice (6 weeks old) came from Guangdong Jiajingda Biotechnology. Prior to the commencement of the experiment, all mice underwent one week of acclimation. During the renal IRI experiments in a sterile operating room, experimenters conducted anesthesia disposal on the mice *via* intraperitoneal injection of a 1% pentobarbital sodium solution. After kidney exposure through bilateral dorsal incisions, we adopted noninvasive arterial clips for clamping bilateral renal arteries for a period of 30 min (no renal clips were applied in the sham group). Following the removal of the arterial clips, the kidneys exhibited a red appearance, indicative of the restored renal blood flow. Subsequently, the mice in cages were subjected to 2, 4, 6, 12, 24, 48, or 72 h of reperfusion. When the experiment ended, experimenters performed anesthesia disposal, and collected blood from the left ventricle for the measurement of serum creatinine (SCr) and blood urea nitrogen (BUN) levels *via* an automated biochemical analyzer (Hitachi, Japan). Phosphate-buffered saline (PBS) was perfused into the mice through the left ventricle until the blood was cleared from the kidneys, after which the kidney tissues were harvested for subsequent experiments.

### 2.6. Histopathology and immunofluorescence analysis

Mouse kidney tissues underwent fixation in 4% paraformaldehyde, dehydration *via* an ethanol gradient, and paraffin-embedding. Three-micrometre-thick sections were prepared in accordance with the producer’s protocol and stained with haematoxylin-eosin (HE) and peroxynitrite Schiff’s stain (PAS). The extent of renal tubular injury was quantitatively assessed *via* ImageJ software. Double immunofluorescence staining for SESN2 (1:100; UpingBio Technology; YP-Ab-12582) was performed, with each antibody incubated separately. Specifically, the sections received one night of culture using the SESN2 primary antibody at 4 °C, and another 1 h of incubation with a fluorescein isothiocyanate (FITC)-conjugated secondary antibody (1:200; Beyotime; A0562) at 37 °C after three times of PBS wash. Nuclear DNA was stained with 4′,6-diamidino-2-phenylindole (DAPI). A Nikon Eclipse Ti2-E fluorescence microscope was employed to capture images. Relevant analysis and quantification were conducted in a blinded manner using the ImageJ software.

### 2.7. Western blot analysis

Extraction of the total protein content of the kidney was conducted *via* a homogenizer in a radioimmunoprecipitation assay (RIPA) buffer that contained 1% phenylmethanesulfonyl fluoride (PMSF). Quantification of protein concentrations relied on a BCA assay kit (Beyotime Inc.). After SDS–PAGE separation, 30 μg of protein were moved onto a 0.45 μm polyvinylidene fluoride (PVDF) membrane (Millipore, Massachusetts, USA) for further analysis. NcmBlot Blocking Buffer (New Cell & Molecular Biotech) was used for the blockage of the nonspecific antigens. Immunoblots were detected using different primary antibodies (dilution 1:1000), followed by HRP-labeled secondary antibodies (dilution 1:5000), with detection performed on the ChemiDoc system (Bio-Rad, Inc., USA). The antibodies used were anti-SESN2, anti-KIM-1, and anti-β-actin (China Protein Technology).

### 2.8. Construction of an animal model of renal IRI induced by S-PPE NP

Healthy male C57BL/6J mice (6 weeks old) with a normal SPF came from Guangdong Jiajingda Biotechnology. All the mice underwent one week of acclimation. prior to the experiment. Experimenters divided mice into four groups (*n* = 6): (1) the healthy control group, (2) the healthy untreated S-PPE NP group, (3) the IRI-24 h model group, and (4) the S-PPE NP-treated IRI group. During renal IRI experiments in a sterile animal operating room, anesthesia was induced *via* the intraperitoneal injection of 1% pentobarbital sodium solution. After kidney exposure by bilateral dorsal incisions, the bilateral renal arteries were occluded *via* the application of noninvasive arterial clips for 30 min (artery clipping was not allowed for the sham group). Following the removal of the arterial clips, a reddening of the kidneys was observed, indicating the restored renal blood flow. The mice in cages then underwent 2 h of reperfusion. Subsequently, groups (2) and (4) were injected with DiD/S-PPE NP (80 mg/kg) through the tail vein, and reperfusion was extended for an additional 24 h. When the experiments ended, experimenters reanesthetized mice, and collected blood samples from the left ventricle to measure the SCr and BUN level *via* an automated biochemistry analyzer (Hitachi). We harvested the hearts, livers, spleens, lungs, and kidneys of each mouse for small-animal imaging to observe the aggregation of particles in each viscera. When the experiments terminated, experimenters took kidney tissues for subsequent analysis in accordance with the procedures outlined in Sections 2.7 and 2.8.

### 2.9. Determination of ROS and apoptosis in renal tissues

Quantification was conducted on ROS level in renal tissues with dihydroethidine (DHE; MCE, USA). Fresh kidney tissues collected after cardiac perfusion were mounted in Optimal Cutting Temperature Compound (OTC, Epson, China). Tissues were sliced into 5-µm-thick sections, which underwent 15 min of incubation using DHE (10 µmol/L) and DAPI (10 µg/mL) after two times of PBS rinses, so as to facilitate counterstaining. Following the incubation period, the sections were rinsed twice with PBS, with buffered glycerol being added dropwise. Subsequently, the coverslips were mounted, and a Zeiss fluorescence microscope (Leica, Germany) was employed to observe the samples. Furthermore, a TUNEL assay kit (Beyotime, China) served for identifying the apoptotic cells in renal tissues, as per the producer’s protocol.

### 2.10. Statistical analyses

Data presentation follows the mean ± standard deviation (SD) format. The data were processed, analyzed statistically and plotted using the R software, version 4.2.2, Figdraw (https://www.figdraw.com), and ProcessOn (https://www.processon.com), respectively. Statistical analyses included two-sided unpaired Student’s t test and two-way ANOVA. *p* < 0.05 indicated statistical significance.

## 3. Results

### 3.1. Screening of differential genes associated with SESN2 and functional enrichment analysis

**Figure 1.**
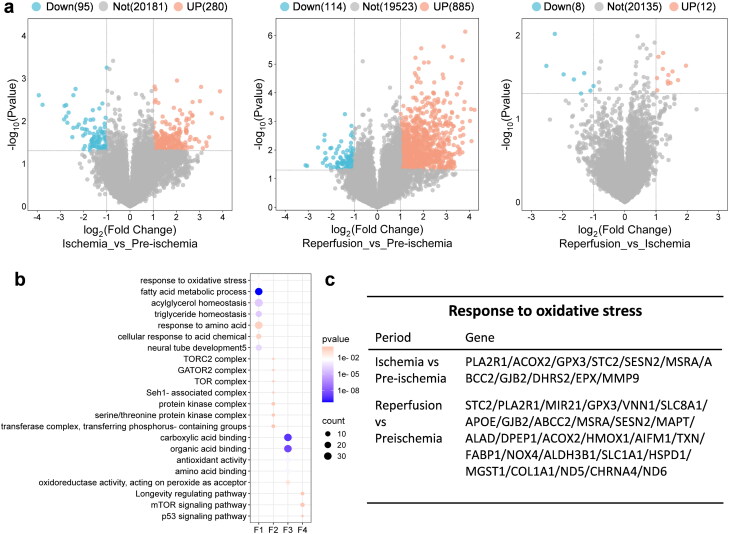
DEGs and functional enrichment analysis before, during, and after ischemia. (a) Volcano plots of DEGs generated *via* the DESeq2 method: ischemia vs. preischemia, postischemia vs. preischemia, and postischemia vs. ischemia (from left to right). (b) bubble plots showing gene ontology and Kyoto Encyclopedia of Genes and Genomes (KEGG) enrichment analysis associated with SESN2: F1–4 for biological process (BP), cellular component (CC), and molecular function (MF), and KEGG pathway enrichment, respectively. (c) DEGs involved in the oxidative stress response. DEGs: Differentially expressed genes.

Differential gene expression analyses were conducted on preischemic, ischemic, and postischemic kidney samples using the DESeq2 method, which identified 355, 999, and 20 DEGs, respectively (Figure 1a). Based on GO and KEGG enrichment results, pathways associated with SESN2 were identified (Figure 1b), which are involved in the regulation of intracellular ROS and fatty acid metabolism, including the mTOR and P53 pathways. Genes linked to oxidative stress were identified at each stage (Figure 1c). The data indicated an increase in genes related to oxidative stress during the postischemic reperfusion phase compared to the ischemic phase, with SESN2 primarily active during the postischemic stage. Figure 1 illustrates the existing knowledge regarding the role of oxidative stress in IRI.

### 3.2. Exploring the effect of S-PPE NP on SESN2 expression during H/R injury in vitro

**Figure 2.**
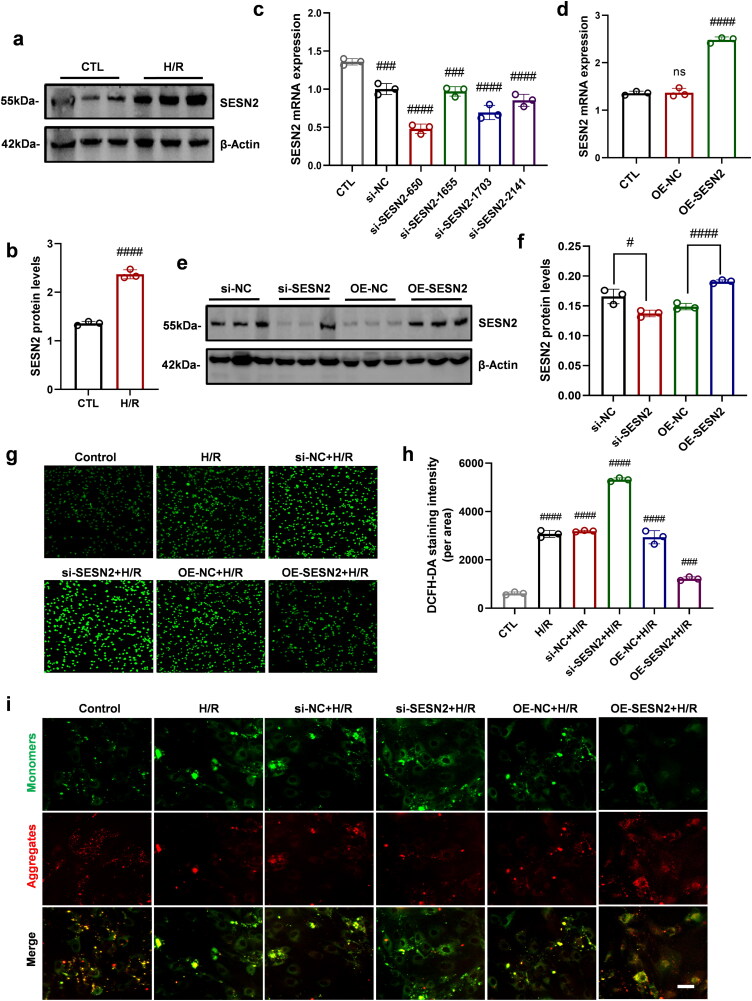
Increased expression of SESN2 in cellular H/R model, a protective factor against H/R injury. (a) WB detection of SESN2 expression in the H/R model of HK-2 cells, (b) and its gray-scale statistical graph. (c,d) qPCR and (e) WB detection of SESN2 knockdown and overexpression effects (f) and their WB grayscale statistical graphs. (g) Effect on ROS homeostasis in the H/R model after interfering with SESN2 expression, (h) Statistical analysis of mean fluorescence intensity. (i) Effect of SESN2 silencing on mitochondrial homeostasis in the H/R model. Scale bar: 100 μm (f) and 20 µm (h); magnification: 200×; *n* = 3. #, *p* < 0.05; ##, *p* < 0.005; ###, *p* < 0.001; ####, *p* < 0.0001 vs. Ctl. H/R, hypoxia/reoxygenation; SESN2, Sestrin2; β-actin, actin beta.

Initially, an H/R model was established in HK-2 cells, and SESN2 expression was analyzed in both control and H/R groups *via* WB (Figure 2a,b). SESN2 expression was modulated using si-SESN2 RNA and an SESN2 overexpression plasmid; its effects were evaluated through qPCR and WB analysis. Results showed that si-SESN2-650 markedly reduced SESN2 expression, while the overexpression plasmid significantly enhanced SESN2 levels (Figure 2c–f). Intracellular ROS levels were quantified using a DCFH-DA probe, revealing that low SESN2 expression increased ROS levels and aggravated injury in the H/R model, whereas SESN2 overexpression reduced ROS levels and mitigated injury (Figure 2g,h). Mitochondrial membrane potential was assessed using a JC-1 probe; low SESN2 expression decreased mitochondrial membrane potential, whereas SESN2 overexpression maintained mitochondrial homeostasis by increasing mitochondrial membrane potential (Figure 2i). In summary, these results demonstrated that SESN2 expression levels are closely linked to the severity of oxidative stress and that SESN2 acts as a protective factor against H/R injury.

### 3.3. Examination of the role of SESN2 in the H/R model and its relationship with S-PPE NP

**Figure 3.**
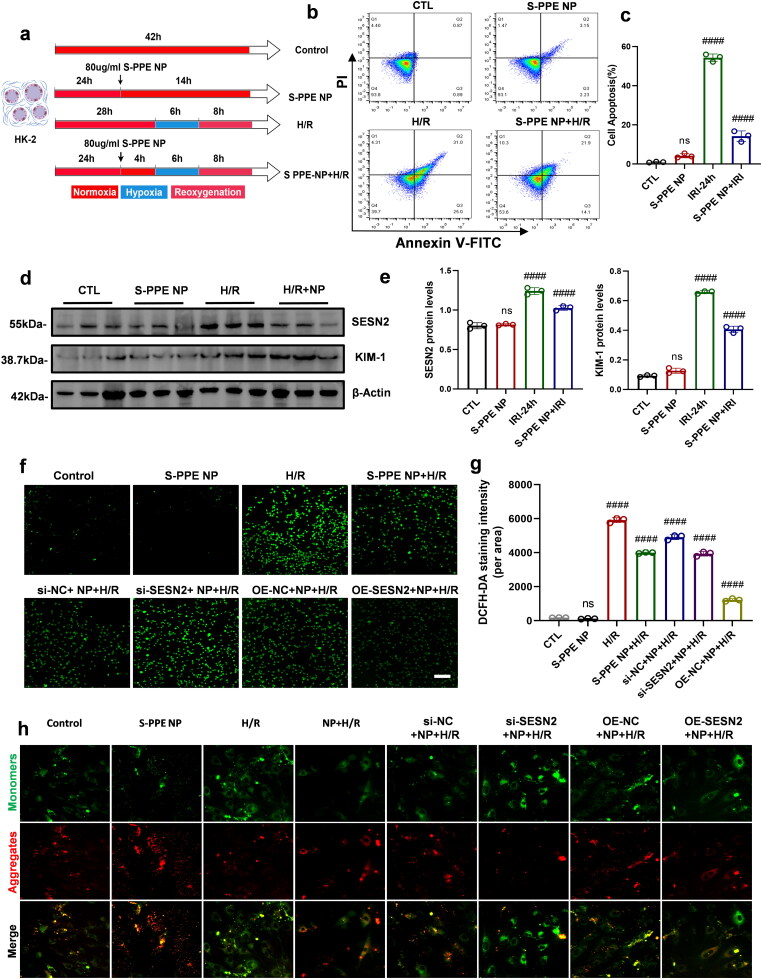
Expression of SESN2 alters the antioxidant effect of S-PPE NP. (a) Schematic representation of IRI modeling in HK-2 cells. (b) Apoptotic state of IRI cells after S-PPE NP treatment. (c) Statistical analysis of the level of apoptosis in cells. (d) Expression levels of related proteins and (e) their corresponding grayscale analysis. (f) Effect of S-PPE NP on ROS scavenging capacity after interfering with SESN2 expression. (g) Statistical analysis of mean fluorescence intensity. (h) Effect of SESN2 silencing on mitochondrial homeostasis in the H/R model of S-PPE NP treatment. Scale bar: 100 μm (f) and 20 µm (h); magnification: 200×; *n* = 3; #, *p* < 0.05; ##, *p* < 0.005; ###, *p* < 0.001; ####, *p* < 0.0001 vs. Ctl. H/R, hypoxia/reoxygenation; SESN2, Sestrin2; KIM-1, kidney injury molecule 1; β-actin, beta-actin.

Initially, an H/R model with S-PPE NP treatment was established in HK-2 cells (Figure 3a), and apoptosis was assessed using the annexin V-FITC/PI method. The results indicated a significant increase in apoptosis in the H/R model group, whereas apoptosis was reduced in the S-PPE NP-treated group, suggesting a therapeutic effect of S-PPE NP on cellular H/R damage (Figure 3b,c). Subsequently, WB analysis revealed increased SESN2 expression and elevated levels of the kidney injury molecule KIM-1 in the H/R model, which were reduced following S-PPE NP treatment (Figure 3d,e). Intracellular ROS and mitochondrial membrane potential levels were quantified using DCFH-DA and JC-1 probes, respectively. The results demonstrated that low SESN2 expression diminished the scavenging capacity of S-PPE NP for reactive oxygen species and disrupted mitochondrial homeostasis, while overexpression of SESN2 had the opposite effect (Figure 3f–h). In conclusion, SESN2 expression levels were closely associated with the antioxidative stress capability of S-PPE NP, indicating that SESN2 may be an effector of S-PPE NP action.

### 3.4. Inspection of the SESN2/AMPK/KIM-1 pathway

**Figure 4.**
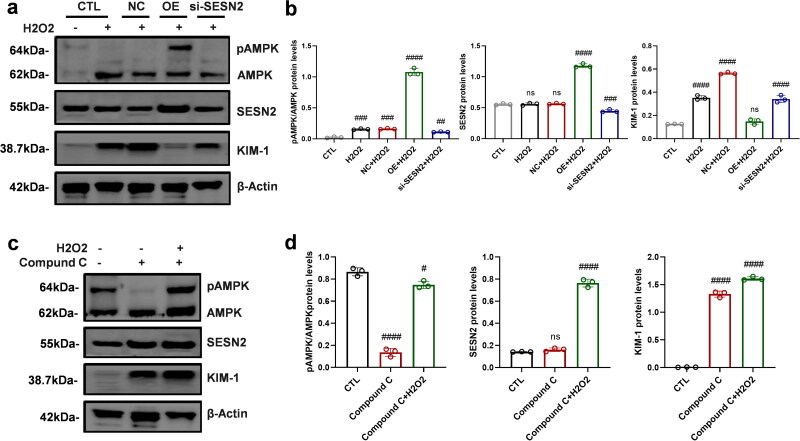
Validation of the SESN2/AMPK/KIM-1 pathway.Under H2O2 environment, (a) WB detection of the effect of perturbation of SESN2 expression on AMPK phosphorylation, KIM-1 expression levels (b) and gray level statistics of related proteins. (c) WB detection of the effect of AMPK inhibitor compound C on SESN2 and KIM-1 expression, (d) and gray value statistics of related proteins. *n* = 3; #, *p* < 0.05; ##, *p* < 0.005; ###, *p* < 0.001; ####, *p* < 0.0001 vs. Ctl. H/R, hypoxia/reoxygenation; AMPK, Adenosine 5’-monophosphate (AMP)-activated protein kinase; SESN2, Sestrin2; KIM-1, kidney injury molecule 1, β-actin, beta-actin.

Initially, an H_2_O_2_ model was established to simulate the oxidative stress environment, and SESN2 expression was modulated by transfecting si-SESN2 and SESN2 overexpression vectors. The phosphorylation of AMPK and the expression of KIM-1 were assessed by WB (Figure 4a,b). Results indicated that SESN2 overexpression increased AMPK phosphorylation and decreased KIM-1 expression, while SESN2 inhibition decreased AMPK phosphorylation and increased KIM-1 expression (Figure 4c,d). These results suggested that alterations in SESN2 expression could induce changes in AMPK phosphorylation and KIM-1 expression, establishing AMPK and KIM-1 as downstream effectors of SESN2. The administration of Conqueror C inhibited AMPK phosphorylation and increased KIM-1 expression but did not affect SESN2 expression, supporting the plausibility of the SESN2/AMPK/KIM-1 pathway.

### 3.5. Construction of a mouse renal IRI model and evaluation of tissue damage over time

**Figure 5.**
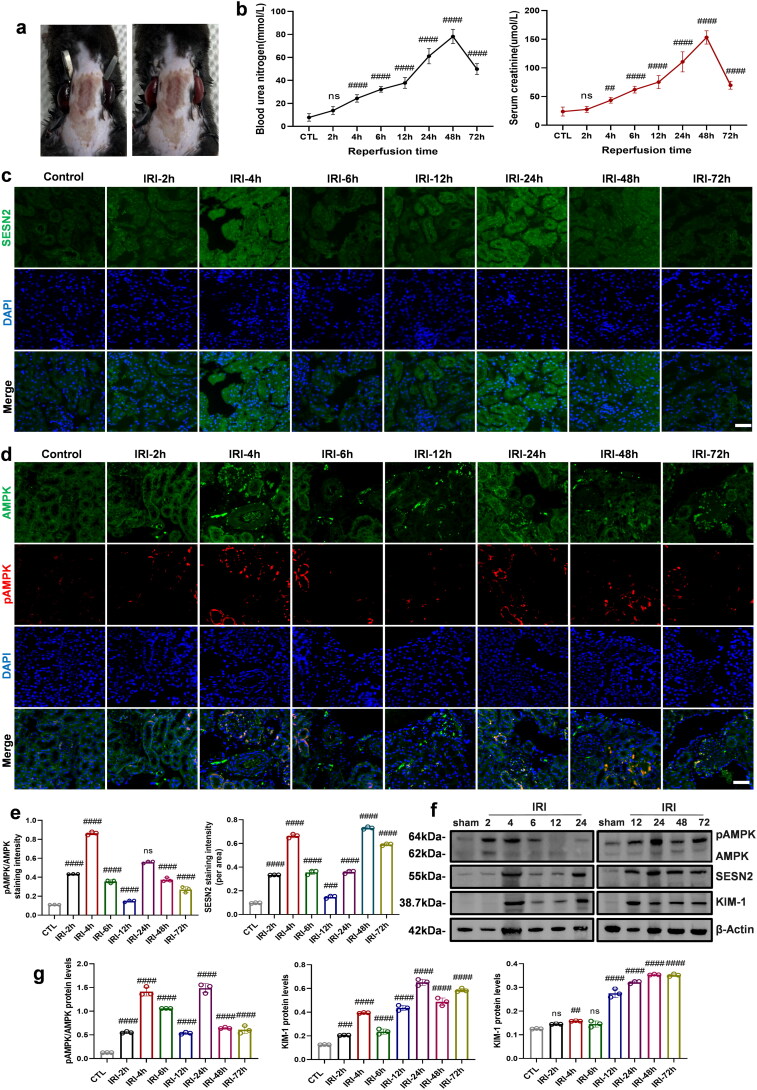
Damage status at different time points in the mouse IRI model. (a) Representative mouse IRI model. (b) Serum creatinine (SCr) and blood urea nitrogen (BUN) levels at differenttime points after IRI. (c) SESN2 expression and (d) AMPK phosphorylation in tissues at different time points after IRI were detected by immunofluorescence and (e) statistical analysis of average fluorescence intensity of corresponding proteins. (f) Western blot analysis of SESN2, KIM-1 expression and AMPK phosphorylation levels at different time points in the IRI model and (g) corresponding statistical analysis of grayscale values. Scale bar: 20 µm; magnification: 1000×; *n* = 6 mice per group; #, *p* < 0.05; ##, *p* < 0.005; ###, *p* < 0.001; ####, *p* < 0.0001 vs. Ctl. IRI, ischemia‒reperfusion injury; AMPK, Adenosine 5’-monophosphate (AMP)-activated protein kinase; SESN2, Sestrin2; KIM-1, kidney injury molecule 1; β-actin, actin beta.

Initially, a mouse IRI model was established (Figure 5a), and changes in SCr and BUN were monitored over time. SCr and BUN levels increased initially, peaked at 48 h, and then gradually decreased (Figure 5b). To further investigate SESN2 expression and its correlation with the severity of renal injury, double immunofluorescence staining was performed. SESN2 expression exhibited a bimodal pattern, peaking at 4 h, decreasing, then peaking again at 24 h before gradually declining. The AMPK phosphorylation levels mirrored the changes in SESN2 expression (Figure 5c–e). WB analysis confirmed these findings and showed that KIM-1 expression increased over time, which supported the potential regulatory role of the SESN2 protein during the injury process (Figure 5f,g).

### 3.6. Selection of 24 h as the modeling time for studying the regulatory mechanism of S-PPE NP on SESN2

**Figure 6.**
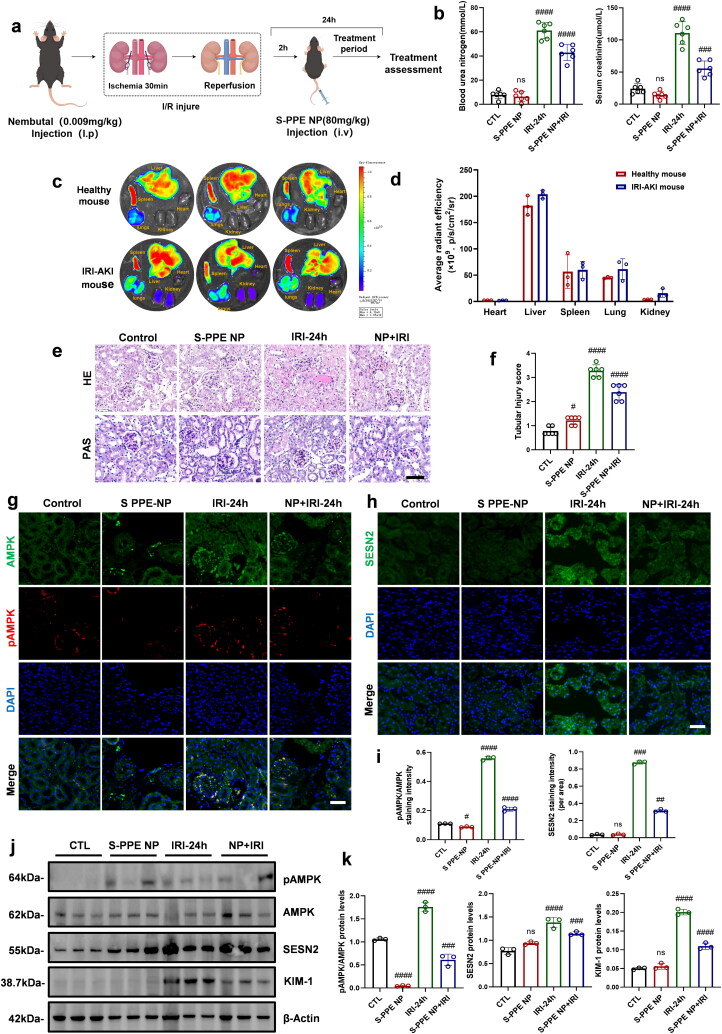
Evaluation of the therapeutic efficacy of S-PPE NP in treating AKI in mice. (a) Treatment of AKI mice with S-PPE NP. (b) Serum creatinine and blood urea nitrogen levels after S-PPE NP treatment. (c) Accumulation of DiD-S-PPE NP in various organs of IRI mice. (d) Statistical analysis of the fluorescence intensity of NP particles in different organs. (e) PAS and HE staining of animal tissues after S-PPE NP treatment. (f) Statistical analysis of kidney injury scores in mice. Immunofluorescence staining of (g) SESN2 and (h) phosphorylated AMPK in mouse tissues after S-PPE NP treatment. (i) Fluorescence intensity analysis of the stained tissues. (j) Western blot analysis of protein expression levels. (k) Grayscale analysis of the bands. Scale bar: 20 µm; magnification: 1000×; *n* = 6 mice per group; #, *p* < 0.05; ##, *p* < 0.005; ###, *p* < 0.001; ####, *p* < 0.0001 vs. Ctl. IRI, ischemia‒reperfusion injury; DiD,1,1′-dioctadecyl-3,3,3’,3′-tetramethylindodicarbocyanine,4-chlorobenzenesulfonate salt; PAS, periodic acid‒Schiff; HE, hematoxylin‒eosin; AMPK, Adenosine 5’-monophosphate (AMP)-activated protein kinase; SESN2, Sestrin2; KIM-1, kidney injury molecule 1; β-actin, actin beta.

A mouse model of IRI treated with S-PPE NP was established (Figure 6a). Urea nitrogen and serum creatinine (SCr) levels were quantified, showing a reduction in SCr and urea nitrogen levels following S-PPE NP treatment compared to the IRI group (Figure 6b). Small-animal imaging was used to track S-PPE NP accumulation in various organs, revealing significant accumulation in the liver and increased deposition in the kidneys after IRI, suggesting transport of NPs to the lesion site to exert functional effects (Figure 6c,d). Histological analysis using HE and PAS staining demonstrated that S-PPE NP treatment ameliorated renal injury in IRI (Figure 6e,f). Immunofluorescence showed increased SESN2 expression and AMPK phosphorylation in IRI compared to controls, which were reduced by S-PPE NP treatment (Figure 6g–i). WB results corroborated the immunofluorescence findings, showing a correlation between KIM-1 expression and AMPK phosphorylation levels (Figure 6j,k).

### 3.7. Diagram of interaction mechanisms

**Figure 7.**
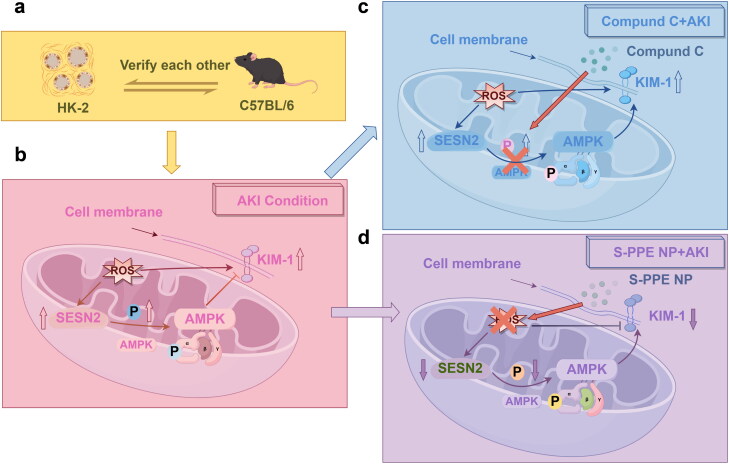
Diagram of interaction mechanisms. We established two models of acute kidney injury using HK-2 cells *in vitro* and C58BL/J mice *in vivo* to mutually validate the mechanism of SESN2-mediated oxidative stress action (a). It was divided into three main parts: (1) to detect the expression of SESN2, KIM-1 and pAMPK under AKI conditions (b); (2) to detect the expression of SESN2 and KIM-1 after the administration of compound C to inhibit pAMPK and to clarify the upstream and downstream of the pathway (c); and (3) the effect of S-PPE NP treatment on the expression of SESN2, KIM-1, pAMPK expression and to analyze the mechanism of NP treatment for AKI (d).

Two models of AKI, *in vitro* in HK-2 cells and *in vivo* in C58BL/J mice, were established to validate the mechanism of SESN2-mediated oxidative stress response (Figure 7a). The study was divided into three parts: (1) SESN2, KIM-1, and pAMPK expression increased under AKI conditions (Figure 7b); (2) Administration of Compound C following pAMPK treatment inhibited KIM-1 expression without affecting SESN2, indicating that SESN2 facilitates kidney repair by activating AMPK phosphorylation, thereby reducing KIM-1 expression (Figure 7c); (3) S-PPE NP treatment alleviated kidney tissue and cell injury by scavenging reactive oxygen species and reducing oxidative stress stimulation of the SESN2/AMPK/KIM-1 pathway, resulting in lower SESN2, KIM-1, and pAMPK expression compared to the untreated model (Figure 7d).

## 4. Discussion

In clinical practice, identifying therapeutic markers for AKI is crucial for diagnosis and monitoring therapeutic response [17]. Our study demonstrates that SESN2 is not only a viable therapeutic target for renal IRI but also responds to the extent of kidney damage, similarly to the kidney injury molecule KIM-1. AKI induces increased expression of the stress response protein SESN2 and KIM-1, involving the SESN2/AMPK/KIM-1 pathway. This pathway enables SESN2 to act as an early protective factor, activating the post-injury repair response in renal tissues and cells by promoting AMPK phosphorylation, thereby decreasing KIM-1 expression. Additionally, S-PPE NP directly scavenges ROS, reducing oxidative stress stimulation of SESN2 and KIM-1 and inhibiting their expression. Interference with SESN2 expression affected the efficacy of S-PPE NP, suggesting that SESN2 plays a role in the treatment of IRI-AKI with S-PPE NP and demonstrating the indirect regulatory effect of S-PPE NP on SESN2.

SESN2, the focus of this study, has shown a protective role in IRI across various organs. For instance, preconditioning with gallic acid (GA) and ruthenic acid (OA) attenuated liver IRI through a mechanism mediated by the HO-1/SESN2 pathway [18–20]. Additionally, SESN2 significantly influenced the pathogenesis of myocardial ischemia and attenuated reperfusion injury in studies by Liu et al. [21] and Quan et al. [22]. During renal IRI, SESN2 is upregulated in renal tubules [23,24], suggesting that this pathway could be a useful target for renal injury treatment [25,26]. This is consistent with our experimental results, which confirm SESN2 as a protective factor against kidney injury and link SESN2 to KIM-1 *via* AMPK, revealing the novel SESN2/AMPK/KIM-1 pathway and offering a new idea for IRI-AKI treatment.

In conclusion, S-PPE NP treatment influences SESN2 expression following kidney injury, which in turn regulates AMPK phosphorylation levels and triggers changes in KIM-1 expression. This suggests the existence of a new signaling pathway, SESN2/AMPK/KIM-1, offering a novel perspective on the mechanism of S-PPE NP therapy for kidney disease. Further studies could validate the feasibility of this pathway in animal models, potentially impacting AKI treatment. However, the present study is not without its limitations. Firstly, it did not analyze the effects of interfering with KIM-1 expression on SESN2 and pAMPK. Secondly, it did not demonstrate the interaction between these three proteins using methods such as co-immunoprecipitation (Co-IP). Therefore, further experiments are necessary to confirm this and address it as a key issue for subsequent studies.

## Data Availability

The datasets provided in this study can be obtained in an online repository. Repository names and accession numbers were detailed in the Methods and Materials.
